# ﻿*Indigoferajintongpenensis* (Fabaceae, Papilionoideae, Indigofereae), a new species from Yunnan, southwest China

**DOI:** 10.3897/phytokeys.241.120230

**Published:** 2024-04-09

**Authors:** Lan Yao, Yan Yang, Xue-Li Zhao, Qiu-Ping Wang, Huan-Chong Wang

**Affiliations:** 1 School of Ecology and Environmental Science, Yunnan University, Kunming 650091, Yunnan, China Yunnan University Kunming China; 2 College of Forestry, Southwest Forestry University, Kunming, 650224, Yunnan, China Southwest Forestry University Kunming China; 3 Herbarium of Yunnan University, Kunming 650091, Yunnan, China Herbarium of Yunnan University Kunming China

**Keywords:** Endemism, Indigofereae, Jintongpen Mountain, mat-forming, short inflorescence, taxonomy

## Abstract

*Indigoferajintongpenensis*, a new species of the subfamily Papilionoideae of Fabaceae, is described and illustrated from Yunnan, southwest China. The new species is characterised by having a prostrate habit, flexible stems and branches, as well as spreading, sub-basifixed, asymmetrically 2-branched trichomes covering the entire plant, discoid calyx, and racemose inflorescences 6–8-flowered, short, 1–2 (–3.5) cm in length, apparently shorter than the leaf. A distribution map and comparison of morphological diagnostic characters with its morphologically similar species are provided. Additionally, a preliminary conservation assessment of *I.jintongpenensis* is proposed following IUCN criteria.

## ﻿Introduction

*Indigofera* L. is a legume genus belonging to the tribe Indigofereae of the subfamily Papilionoideae (Schrire et al. 2005; Azani et al. 2017). With approximately 750 species (Schrire et al. 2009), it is the third largest genus in Fabaceae. The genus is mainly distributed in tropical and subtropical regions worldwide with centres of species diversity primarily occurring in Africa (ca. 550 species), the Sino-Himalayan region (ca. 105 species), Australia (ca. 50 species) and the New World (ca. 45 species) (Schrire et al. 2009). Species of *Indigofera* are typically shrubs, but some are small trees, herbaceous perennials or annuals. The genus is characterised by a combination of the presence of medifixed 2-branched trichomes, pulvinate leaves, axillary simple racemes, anthers with appendiculate connective and flowers with an explosive pollen display (Hutchinson 1964; De Kort and Thijsse 1984). The genus includes economically important species with a variety of uses (Gerometta et al. 2020). Notably, *I.tinctoria* L. and *I.suffruticosa* Mill. are the principal sources for production of natural indigo (Zhang et al. 2019).

China harbours a high level of diversity for *Indigofera* species, including many endemics, with the highest species diversity found in the south-western China (Yin et al. 1992; Gao and Schrire 2010). The first comprehensive revision for Chinese *Indigofera* was proposed by Craib (1913), recognising 57 species with 31 newly named. In the Flora Reipublicae Popularis Sinicae, Fang and Zheng (1994) recognised 81 species and nine varieties. In the latest treatment by Gao and Schrire (2010) in the Flora of China, 79 species and nine varieties were accepted, 45 of which are endemic. Recently, Zhao and Gao (2015), Zhao et al. (2020) and Liu et al. (2022) described three additional species of *Indigofera* in southwest China, highlighting the need for continued field exploration and taxonomical research of the genus in this area.

During our recent field surveys in preparation for a taxonomic revision of the genus *Indigofera* of Yunnan Province in southwest China, we collected an intriguing prostrate plant with a densely spreading indumentum on Jintongpen Mountain of Fumin County. Its racemose inflorescences are relatively short, bearing few flowers. After conducting extensive literature surveys and comparison with related specimens, we concluded this plant does not match with any of the previously described species. Therefore, it is described herein as a new species.

## ﻿Materials and methods

The study followed the normal practice of plant taxonomic survey and herbarium taxonomy. Morphological studies of the new species were based on observation of living plants and specimens collected from the type locality. Digital images available at the JSTOR Global Plants (http://plants.jstor.org/) and at the Chinese Virtual Herbarium (http://www.cvh.ac.cn/), as well the collections housed at CDBI, KUN, PE, PYU, XTBG and YUKU were examined and compared with the new species. Relevant taxonomic literature (e.g. Craib (1913); Fang and Zheng (1994); Sun (2006); Gao and Schrire (2009, 2010); Chauhan et al. (2013); Clark et al. (2015); Pignal and De Queiroz (2019)) was consulted. Morphological studies were carried out on dried material under a stereomicroscope (Olympus SZX2, Tokyo, Japan) and measurements were made using a ruler and a metric vernier caliper. Terminology followed Gao and Schrire (2010).

## ﻿Taxonomy

### ﻿ Indigofera jintongpenensis

Taxon classificationPlantae

Huan C.Wang, L.Yao & X.L.Zhao sp. nov.

01352C0A-9473-5548-9813-5220A05F51CC

urn:lsid:ipni.org:names:77339921-1

Figs 1 , 2 , 3 , 4

#### Type.

China. Yunnan Province: Fumin County, Jintongpen Mountain, alt. 2,730 m, in the scrub of the limestone mountains, 10 June 2022, *H. C. Wang et al. FM16943* (Holotype: YUKU!; isotypes: YUKU!)

**Figure 1.**
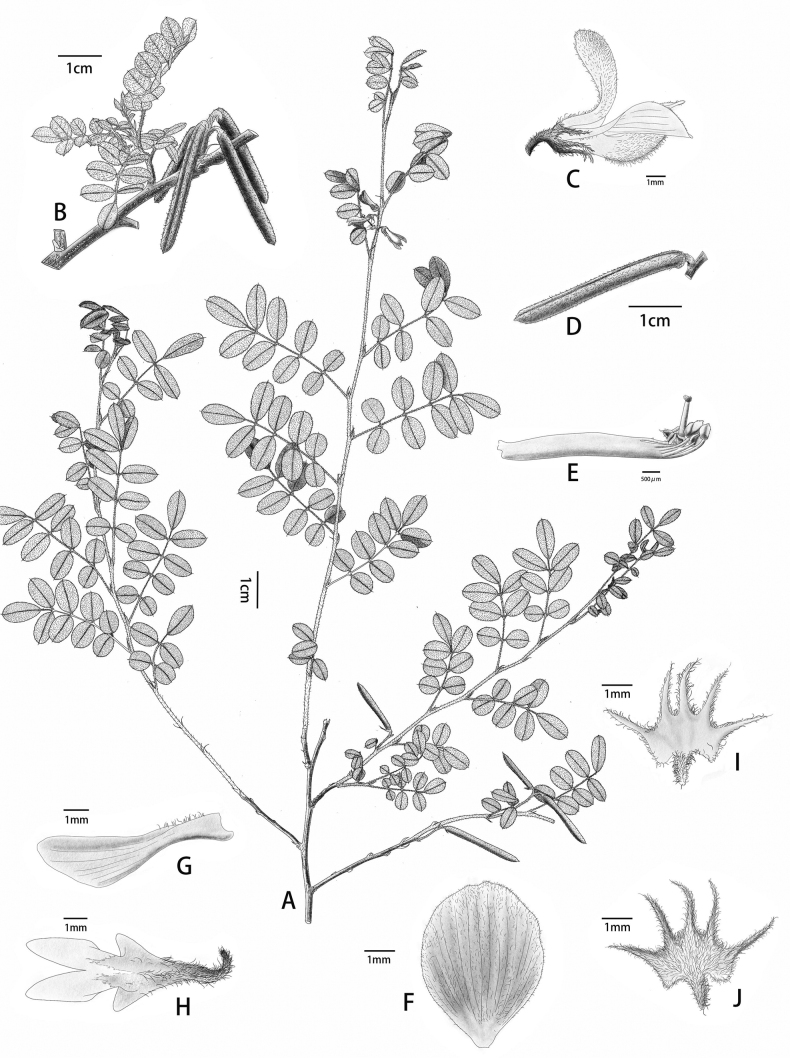
*Indigoferajintongpenensis* sp. nov. **A** habit **B** fruit branch **C** flower (side view) **D** legume **E** style **F** standard **G** wing **H** keel **I** calyx (glabrous inside) **J** calyx (outside with spreading sub-basifixed 2-branched trichomes).

#### Diagnosis.

*Indigoferajintongpenensis* is most morphologically similar to *I.balfouriana* Craib, but it clearly differs from the latter by its habit being prostrate (vs. erect), much-branched stems and branches flexible, leaves usually 7–13-foliolate (vs. 5–9-foliolate), stipules usually 5–7 mm (vs. 3–6 mm) long, inflorescences racemose, 1–2 (–3.5) cm (vs. 2–6 cm) long, 6–8-flowered, legumes 1.5–2.5 cm (vs. 2.5–4.0 cm) long, endocarp not blotched (vs. blotched).

**Figure 2.**
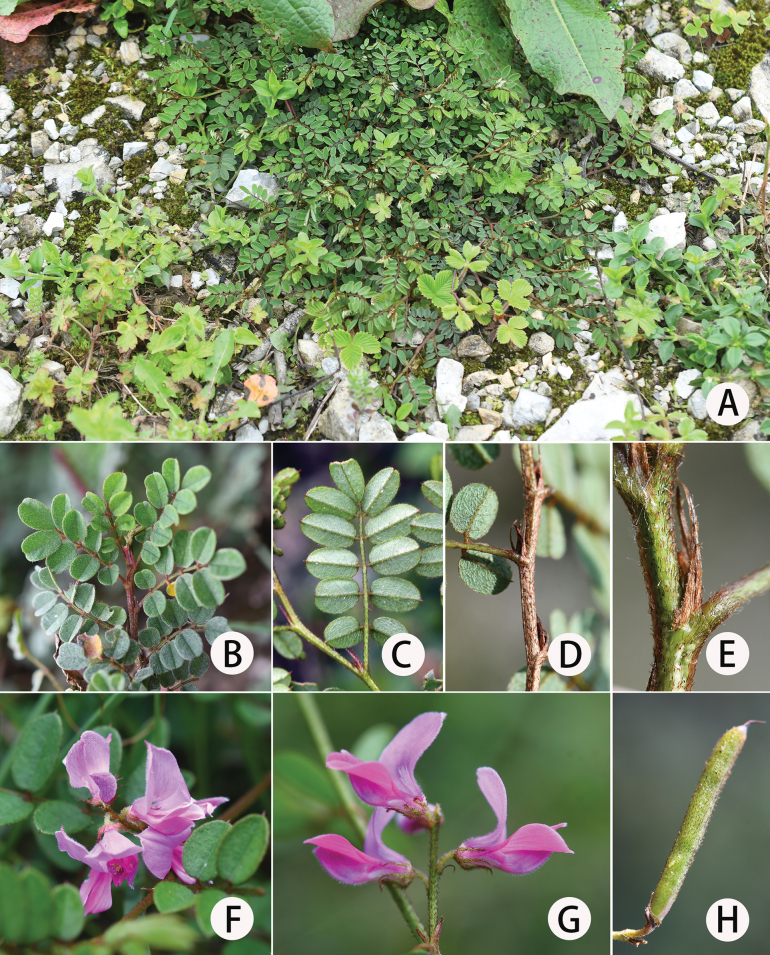
*Indigoferajintongpenensis* sp. nov. **A** habit **B** a portion of branchlet showing adaxial surface of leaflets **C** leaves (abaxial) **D** branchlet **E** stipules **F** leaves and inflorescence **G** inflorescence **H** legume.

#### Description.

Shrubs, prostrate, 10–20 (–30) cm in height. Stems much-branched, terete, slightly 4-angled when young, flexible, usually 1.0–2.5 mm in diameter. Branchlets nearly terete, flexible, 10–20 cm long, with dense spreading white and brown sub-basifixed curly and asymmetrically 2-branched trichomes. Leaves imparipinnate, 2–7 cm × 1.0–2.5 cm, 7–13-foliolate. Stipules narrowly triangular to linear, 5–7 mm long, purple when young, turning brown when old, with spreading, brown, curled and asymmetrically 2-branched trichomes. Petioles 0.2–1.0 cm long, rachis adaxially grooved, with spreading, curled, sub-basifixed, brownish-black, 2-branched trichomes. Leaflets opposite, 0.5–1.5 cm × 0.4–0.8 cm, adaxial surface green, covered with short, spreading, white, 2-branched trichomes, abaxial surface pale green, covered with long, spreading, sub-medifixed, white, 2-branched trichomes and brown, 2-branched trichomes along primary venation; mid-vein abaxially prominent and adaxially impressed, secondary veins inconspicuous; terminal leaflets obovate, apex rounded or retuse, base broadly cuneate; lateral leaflets oblong or elliptic, apex rounded, with a mucro ca. 1 mm long, base rounded or shallowly cordate; petiolules ca. 1 mm long. Inflorescences racemose, 6–8-flowered, axillary, obviously shorter than their subtending leaf, 1–2 (–3.5) cm long. Peduncles 0.2–0.5 cm long; peduncle and rachis densely covered with spreading, white and brown, sub-basifixed, curly and asymmetrically 2-branched trichomes. Bracts narrowly lanceolate to linear, revolute, ca. 2 mm long, caducous. Pedicels ca. 2 mm long, slightly curved, with densely spreading, sub-basifixed, white and brown, 2-branched trichomes. Calyx discoid, spreading, outside with spreading brown and white, sub-basifixed, asymmetrically 2-branched trichomes, glabrous inside; tube ca. 1 mm long; lobes 5, unequal, triangular-lanceolate, ca. 1.5–2.0 mm long, apex long acuminate. Corolla pink; standard obovate-elliptic, 7–9 mm × 4–6 mm, apex rounded, base broadly cuneate, outside with white, soft, 2-branched trichomes, margin ciliate; wings narrowly oblong, ca. 8 mm long, ca. 2 mm wide, base bristly, margin ciliate; keel petals 7–9 mm × ca. 1.5 mm, outside covered with spreading white trichomes towards apex, with spur ca. 1 mm long. Stamens 10, diadelphous, 9 stamens fused and the vexillary one free, 5–6 mm long; anthers ovoid, apically convex. Ovary hairy; style glabrous. Legume cylindrical, sutures thickened, 1.5–2.5 cm × ca. 0.2 cm, apex beaked, with white and brown, medifixed, symmetrically 2-branched trichomes, endocarp not blotched. Seeds 5–7 per legume, oblong to rectangle, 1–2 mm × ca. 1 mm, transverse septa present between seeds.

**Figure 3.**
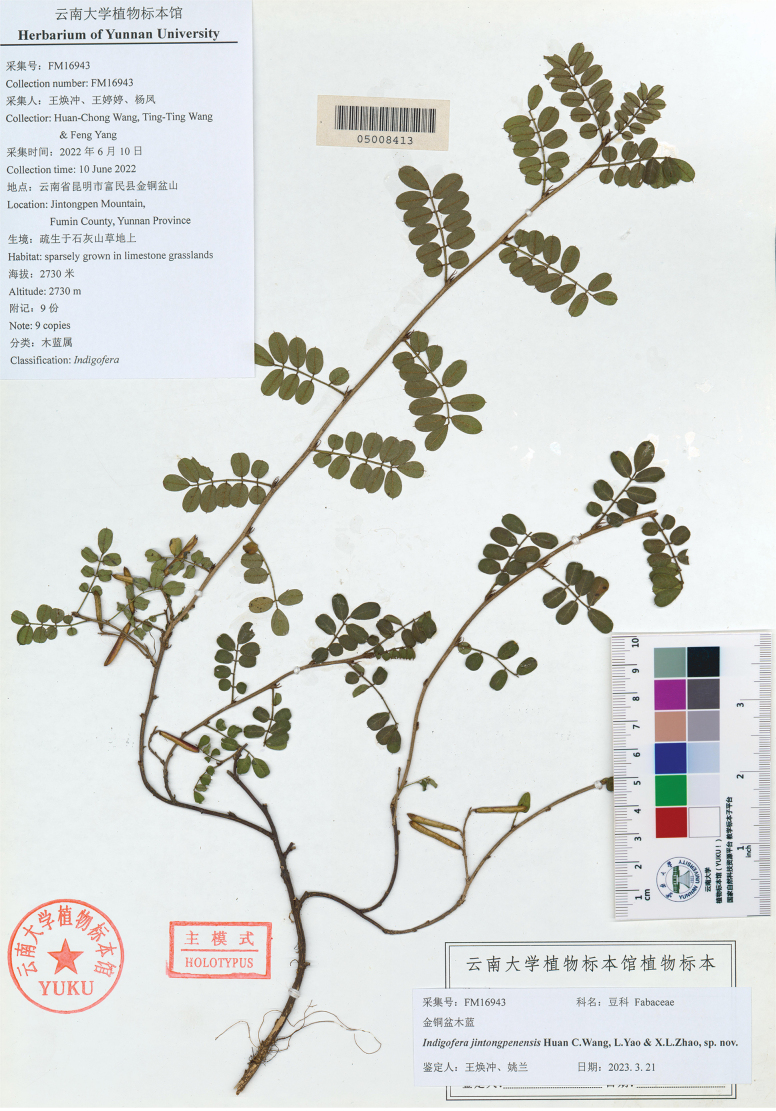
Holotype of *Indigoferajintongpenensis* (YUKU-05008413).

#### Phenology.

Flowering from June to September, fruiting from August to December.

**Figure 4.**
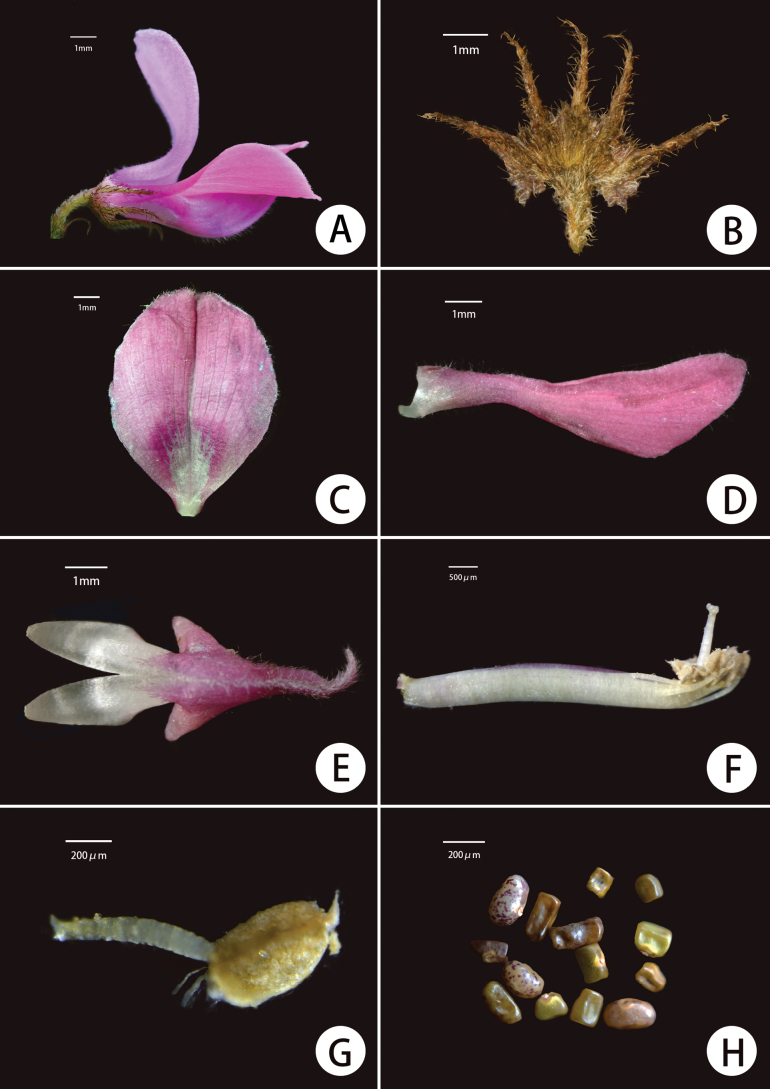
*Indigoferajintongpenensis* sp. nov. **A** flower (lateral view) **B** calyx **C** standard **D** wing **E** keel **F** pistil and stamens **G** stamen **H** seeds.

#### Etymology.

The specific epithet “*jintongpenensis*” is derived from the type locality of the new species, the Jintongpen Mountain and the Latin suffix -*ensis*, indicating the place of origin or growth.

#### Distribution and ecology.

**Figure 5.**
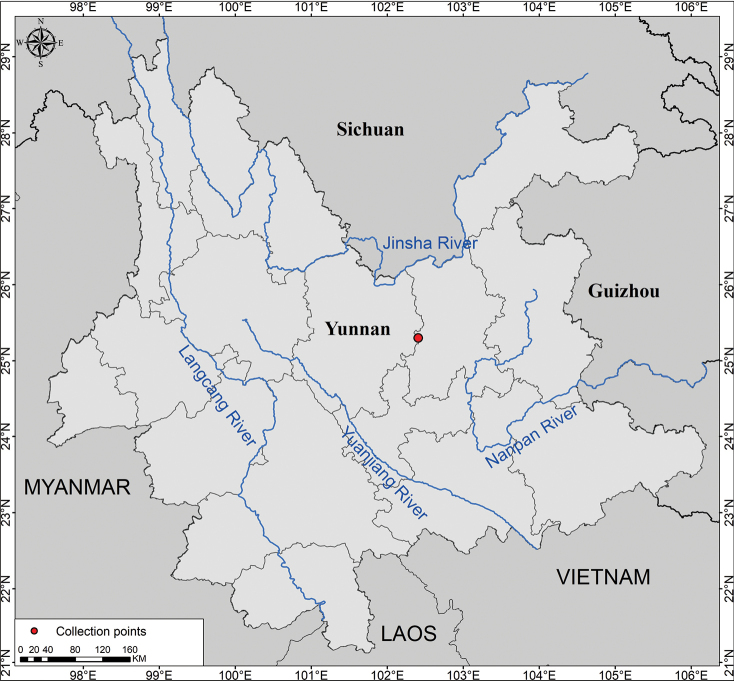
Geographical distribution of *Indigoferajintongpenensis* sp. nov. (red dot).

According to the present investigations, *Indigoferajintongpenensis* is only found in its type locality, the Jintongpen Mountain of Fuming County, located in central Yunnan Province, southwest China (Fig. 5). With a maximum altitude of 2,817 m, the Jintongpen Mountain is the highest peak in Fuming County. *I.jintongpenensis* has been observed at elevations ranging from 2600–2817 m in the summit region of the mountain. It usually grows in the limestone scrub and its association includes *Alliumwallichii* Kunth (Amaryllidaceae), *Asparagusfilicinus* Buch.-Ham. ex D.Don (Asparagaceae), *Berberiswilsoniae* Hemsley (Berberidaceae), *Buddlejamyriantha* Diels (Scrophulariaceae), *Impatiensyaoshanensis* K.M.Liu & Y.Y.Cong (Balsaminaceae), *Loliumperenne* Linn. (Poaceae), *Quercuspannosa* Hand.-Mazz. (Fagaceae), *Quercusrehderiana* Handel-Mazzetti (Fagaceae), *Silenegracilicaulis* C.L.Tang (Caryophyllaceae) and so on. It can also be found occasionally under the thickets predominated by *Quercuspannosa* Hand.-Mazz. (Fagaceae).

#### Conservation status.

*Indigoferajintongpenensis* is a rare species with a restricted distribution and small population size. It is only known from a single locality on the Jintongpen Mountain in the Fumin County, southwest China, which is not within any protected area. The estimated area of occupancy (AOO) is less than 20 km^2^. The total population size is estimated to be fewer than 250 mature individuals. Following the IUCN criteria (IUCN 2022), this new species should be classified as Endangered [EN (B2abiii, D)].

#### Taxonomic notes.

*Indigoferajintongpenensis* is mainly characterised by having a prostrate habit, 2-branched trichomes that are spreading and asymmetrical, and racemose inflorescences that are 6–8-flowered and relatively short (mostly 1–2 cm in length). It is most morphologically similar to *I.balfouriana* Craib in terms of indumentum on various parts of the plant, as well as flower shape and size, but is clearly distinguished by the features pointed out in the diagnosis as well as others (Table 1).

**Table 1.** Morphological comparison amongst *Indigoferajintongpenensis* and the related species.

Character	*I.jintongpenensis*	*I.balfouriana*	*I.szechuensis*
Habit	prostrate shrub	erect shrub	erect shrub
Plant height (m)	0.1–0.2 (–0.3)	0.6–2.0	0.8–2.5
Stems	prostrate	erect	erect
Stem indumentum	spreading, sub-basifixed curly and asymmetrical	spreading or subspreading appressed or 2-branched	appressed mixed and medifixed symmetrically
Leaf length (cm)	2–7	3–9	4–10
Stipule size (mm)	5–7	3–6	up to 2.5
No. of leaflets	7–13	5–9	(5–) 7–13
Leaflet size (mm)	5–15 × 4–8	6–20 × 4–13	5–20 × 4–9
Raceme length (cm)	1–2 (–3.5)	2–6	10–19
Peduncle size (cm)	0.2–0.5	0.5–1.5	0.8–2.7
Calyx shape	discoid	bell-shaped	cup-shaped
Colour of corolla	pink	red to purple	crimson-red
Standard size (mm)	7–9 × 4–6	6.0–9.5 × 5.0–6.0	7.5–9.5 × 5.0–6.5
Legume length (cm)	1.5–2.5	2.5–4.0	3.5–4.0

*Indigoferajintongpenensis* is also similar to *Indigoferaszechuensis* Craib in overlapping leaf length, the number of leaflets and overlapping standard size (Table 1). However, *I.szechuensis* differs from *I.jintongpenensis* in having an erect habit, 2-branched trichomes appressed, symmetrical and medifixed, stipules 2.5 mm long, inflorescences 10–19 cm long, peduncles 8–27 mm long (Table 1).

#### Additional specimens examined

**(Paratypes).** China. Yunnan: Fumin County, Jintongpen Mountain, alt. 2770 m, 6 September 2023, *H. C. Wang et al. FM22978*, *FM22988*, *FM23008* (YUKU); Fumin County, Jintongpen Mountain, alt. 2710 m, 2 November 2023, *H. C. Wang et al. FM23540* (YUKU).

## Supplementary Material

XML Treatment for Indigofera jintongpenensis

## ﻿Additional information

### Author ORCIDs

Lan Yao https://orcid.org/0009-0001-4326-2663

Yan Yang https://orcid.org/0009-0001-0764-8745

Xue-Li Zhao https://orcid.org/0000-0002-8773-5602

Qiu-Ping Wang https://orcid.org/0000-0001-7765-4174

Huan-Chong Wang https://orcid.org/0000-0001-8562-8849

### Data availability

All of the data that support the findings of this study are available in the main text.
